# Familial intragenic duplication of *ANKRD11* underlying three patients of KBG syndrome

**DOI:** 10.1186/s13039-015-0126-7

**Published:** 2015-03-26

**Authors:** Milena Crippa, Daniela Rusconi, Chiara Castronovo, Ilaria Bestetti, Silvia Russo, Anna Cereda, Angelo Selicorni, Lidia Larizza, Palma Finelli

**Affiliations:** Medical Cytogenetics and Molecular Genetics Lab, IRCCS Istituto Auxologico Italiano, via Ariosto 13, Milano, 20145 Italy; Department of Medical Biotechnology and Translational Medicine, Università degli Studi di Milano, via Viotti 3/5, Milano, 20133 Italy; U.O.S Clinical Genetics and Pediatrics, MBBM Foundation San Gerardo Hospital, Via G. Pergolesi, 33, Monza, (MB) 20052 Italy

**Keywords:** KBG syndrome, *ANKRD11* intragenic duplication, Low-level mosaicism, Genotype–phenotype correlations

## Abstract

**Background:**

KBG syndrome, a rare autosomal disorder characterised by distinctive craniofacial and skeletal features and developmental delay, is caused by haploinsufficiency of the *ANKRD11* gene.

**Results:**

Here we describe two siblings with multiple symptoms characteristic of KBG and their mother with a milder phenotype. In the siblings, array-based comparative genomic hybridization (array CGH) identified an intragenic microduplication affecting *ANKRD11* that was absent from the parents’ array CGH profiles. Microsatellite analysis revealed the maternal origin of the rearrangement and interphase fluorescent *in situ* hybridization (i-FISH) experiments identified the rearrangement in low-level mosaicism in the mother. Molecular characterisation of the duplication allele demonstrated the presence of two mutant *ANKRD11* transcripts containing a premature stop codon and predicting a truncated non-functional protein.

**Conclusions:**

Similarly to deletions and point mutations, this novel pathogenetic rearrangement causes haploinsufficiency of *ANKRD11*, resulting in KBG syndrome.

**Electronic supplementary material:**

The online version of this article (doi:10.1186/s13039-015-0126-7) contains supplementary material, which is available to authorized users.

## Background

KBG syndrome (OMIM 148050) is a rare genetic disorder characterised by distinctive craniofacial features, including oval/triangular face, brachycephaly, hypertelorism, bulbous nasal tip, elongated philtrum, wide and arched eyebrows, macrodontia of the upper central incisors of permanent teeth, skeletal anomalies, hand anomalies, and short stature. Neurological involvement with developmental delay, seizures, and intellectual disability is also observed in affected individuals [1-5]. Since 1975, more than 70 KBG patients have been described [1-16]; among them, a few familial cases have been reported [9,14,15]. This number likely reflects an underestimate, because many features of KBG syndrome have mild clinical presentation, including intellectual disability, and none is prerequisite for the diagnosis [5]. However, based on the review of most reported patients, it has been suggested that at least four out of eight major criteria, namely macrodontia, typical gestalt, neurological anomalies, delayed bone age, costovertebral and/or hand anomalies, short stature, and the presence of an affected first degree relative, should be present for the diagnosis [4].

KBG syndrome is caused by haploinsufficiency of the 16q24.3 *ANKRD11* gene resulting from either heterozygous point mutations [5,12,14-16] or chromosomal microdeletions [6-11,15]. Five patients with deletions encompassing solely the *ANKRD11* gene [6-8], and ten patients with larger deletions including several flanking genes have been described [9,10,14,15]. The *ANKRD11* gene encodes the ankyrin repeat domain-containing protein 11, which is a member of the family of ankyrin repeat domain-containing cofactors that are inhibitors of ligand-dependent transcriptional activation [17]. ANKRD11 has two domains that appear to function in transcriptional repression and one domain that functions in promoting transcription [17]. Recently it has been shown that the abundance of the wild- type ANKRD11 is tightly regulated during the cell cycle; moreover the N-terminus forms homodimers and the ANKRD11 C-terminus is required for protein degradation [16]. All reported mutations described in KBG patients affect a highly conserved domain for transcriptional repression [5,16] and most of them are predicted to lead to premature termination codons, possibly resulting in *ANKRD11* haploinsufficiency [5]. A phenotypic variability among KBG patients has been observed, even if the clinical presentation of mutated and deleted patients is similar, and intra-and interfamilial variation has been observed in both patient groups [15].

Here, we describe a new case of KBG vertical transmission, including two siblings exhibiting typical phenotypes and carrying an *ANKRD11* intragenic duplication, present in low-level mosaicism in their mildly affected mother. This intragenic microduplication, the first rearrangement of its kind reported in this gene, triggers haploinsufficiency of *ANKRD11*, resulting in KBG syndrome and confirming haploinsufficiency as the pathogenetic mechanism underlying this syndrome.

## Case presentation

### Clinical report

The probands are the only children of non-consanguineous Caucasian parents with an unremarkable family history. The boy was born at term by caesarean section after a pregnancy complicated by intrauterine growth restriction (IUGR). Birth parameters: weight, 2,270 g (−2.9 SD); length, 44 cm (−3.5 SD); occipito-frontal circumference (OFC) 31 cm (−3.1 SD); Apgar scores, 9–9. The girl was born at term by caesarean section after an uncomplicated pregnancy. Birth parameters: weight, 2,730 g (−1.8 SD); length 45.5 cm (−2.6 SD); OFC, 35 cm (+0.5 SD); Apgar scores, 9–9. Birth parameter were calculated according to the Italian Neonatal Study (INeS) charts [18]. Both siblings were diagnosed with a congenital atrioventricular canal defect, which was surgically corrected around the age of 2 years.

**Figure 1:**
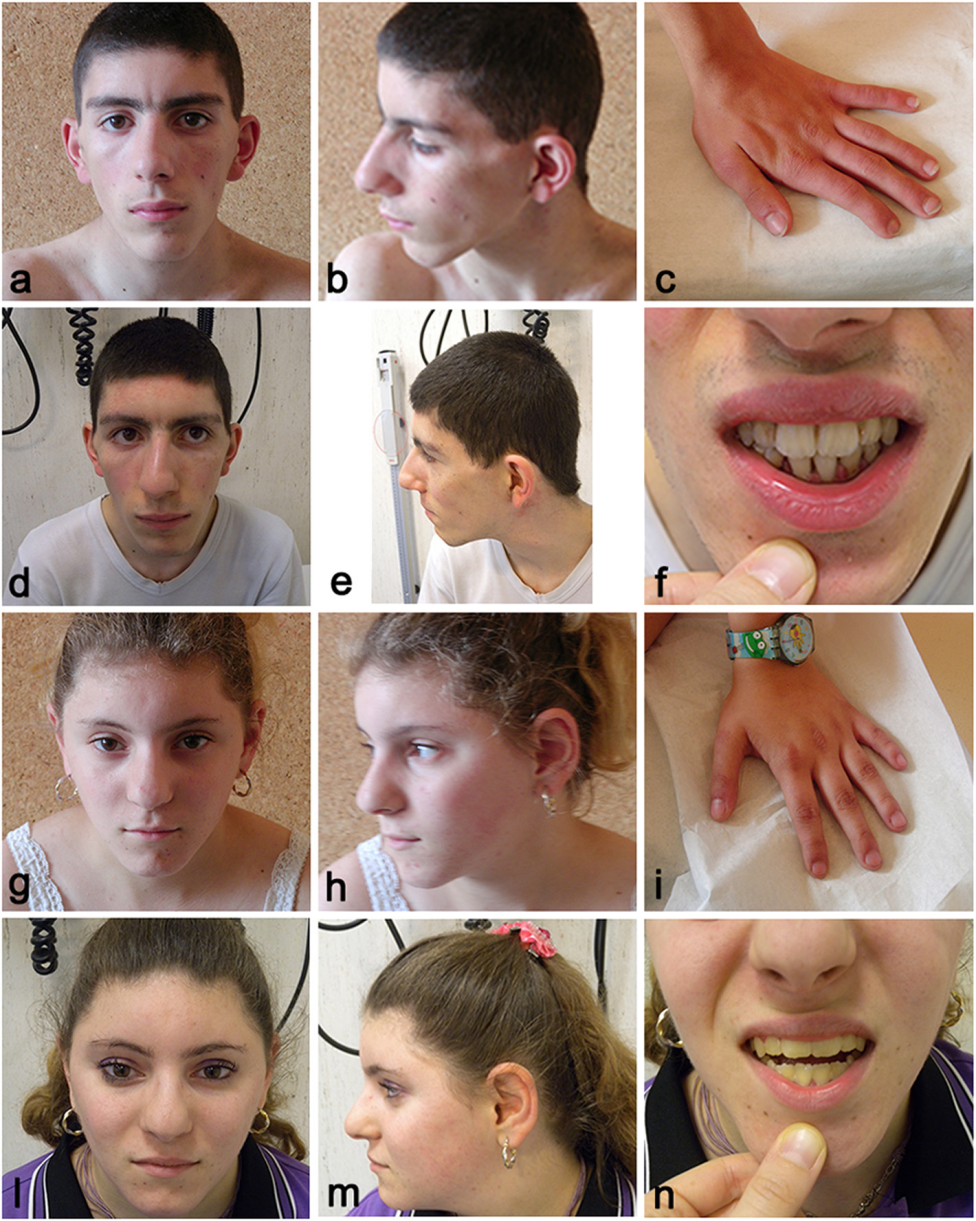
**Facial, dental, and hand profiles of male and female probands.** Frontal **(a, d)** and lateral **(b, e)** views of the male proband at 17 **(a, b)** and 22 **(d, e)** years of age. Frontal **(g, l)** and lateral **(h, m)** views of the female proband at 13 **(g, h)** and 18 **(l, m)** years of age. Facial features comprise a long triangular face, frontal bossing, broad and high nasal bridge with bulbous nasal tip, large ears, macrodontia of central incisors **(f, n)**, retrognathia, and a pointed chin. **(c, i)** Small and short hands were identified in both sibs.

The probands were referred to our centre at the ages of 17 (boy) and 13 (girl) (Figure 1). All growth parameters were below -2 SD [18]. Clinical examination revealed facial dysmorphisms (Figure 1), including long triangular face, frontal bossing, arched and bushy eyebrows (with slight synophrys in the boy), large and prominent ears, broad and high nasal bridge with bulbous nasal tip, anteverted nares, long philtrum, macrodontia of central incisors (Figure 1f and 1n), and a nasal voice. Skeletal anomalies included brachymetacarpia (Figure 1c and 1i). Both patients exhibited moderate intellectual disability. The boy also exhibited third-degree vesicoureteral reflux, and the girl had left ureterocele associated with duplex pelvicalyceal district. The mother exhibited mild facial dysmorphisms, similar to those of her children, and a nasal voice.

### Methods

Array CGH was performed using the Human Genome CGH Microarray Kit 244 K (Agilent Technologies, Palo Alto, CA). For microsatellite analysis, polymorphic loci within 16q24.3 were selected using the UCSC Genome Browser (https://genome.ucsc.edu/), and assays were performed using the AmpliTaqGold kit (Life Technologies, Carlsbad, CA) on an ABI PRISM 310 Genetic Analyzer (Applied Biosystems, Foster City, CA) with a GS ROX500 size standard (Applied Biosystems), separated by POP4, and analysed using GeneScan.

i-FISH analysis was performed on peripheral blood lymphocytes as previously described [19]. The BAC probe CTD-3200N1 (Invitrogen Ltd., Paisley, UK), which maps at 16q24.3, was nick-translation labelled with Cy3-dUTP (Amersham, Chalfont St. Giles, UK). i-FISH false-positive rate was determined on a healthy subject.

For quantitative gene-expression analysis, total RNA was collected using Tempus Blood RNA tubes (Applied Biosystems), isolated using the Tempus Spin RNA Isolation kit (Applied Biosystems), and reverse-transcribed using the High-Capacity cDNA Reverse Transcription kit (Applied Biosystems). Quantitative real-time RT-PCR was performed using an ABI PRISM 7700 Sequence Detection System (Applied Biosystems). Levels of *ANKRD11* mRNA were calculated using the 2-∆∆Ct method, normalized against *GAPDH* (TaqMan ID#: Hs00946576_m1, *ANKRD11* exons 3–4; Hs00946580_m1, *ANKRD11* exons 7–8; Hs00946585_gH, *ANKRD11* exons 12–13; Hs99999905_m1, *GAPDH*; Applied Biosystems). Statistical analysis was performed by two-tailed Student’s t test; *p* < 0.01 was interpreted as significant.

PCR was performed on cDNA using the AmpliTaq Gold® kit (Applied Biosystems) with transcript-specific primers for *ANKRD11* (forward primer: 5′TGGTGAACCTCCTGTTAGGC3′; reverse primer: 5′GAAGCTCTTCCTGCTGTGGT3′; product size: 162 bp, Ta: 59.3°C). Amplicons were sequenced using the Big Dye® Terminator v.3.1 Cycle Sequencing kit (Applied Biosystems). Duplication junction sequences were aligned to the human reference genome sequence (human genome assembly GRCh37/hg19), analysed with the ChromasPro 1.5 software (Technelysium Pty Ltd., Tewantin QLD, Australia), and submitted to GenBank (http://www.ncbi.nlm.nih.gov/WebSub).

### Results

**Figure 2:**
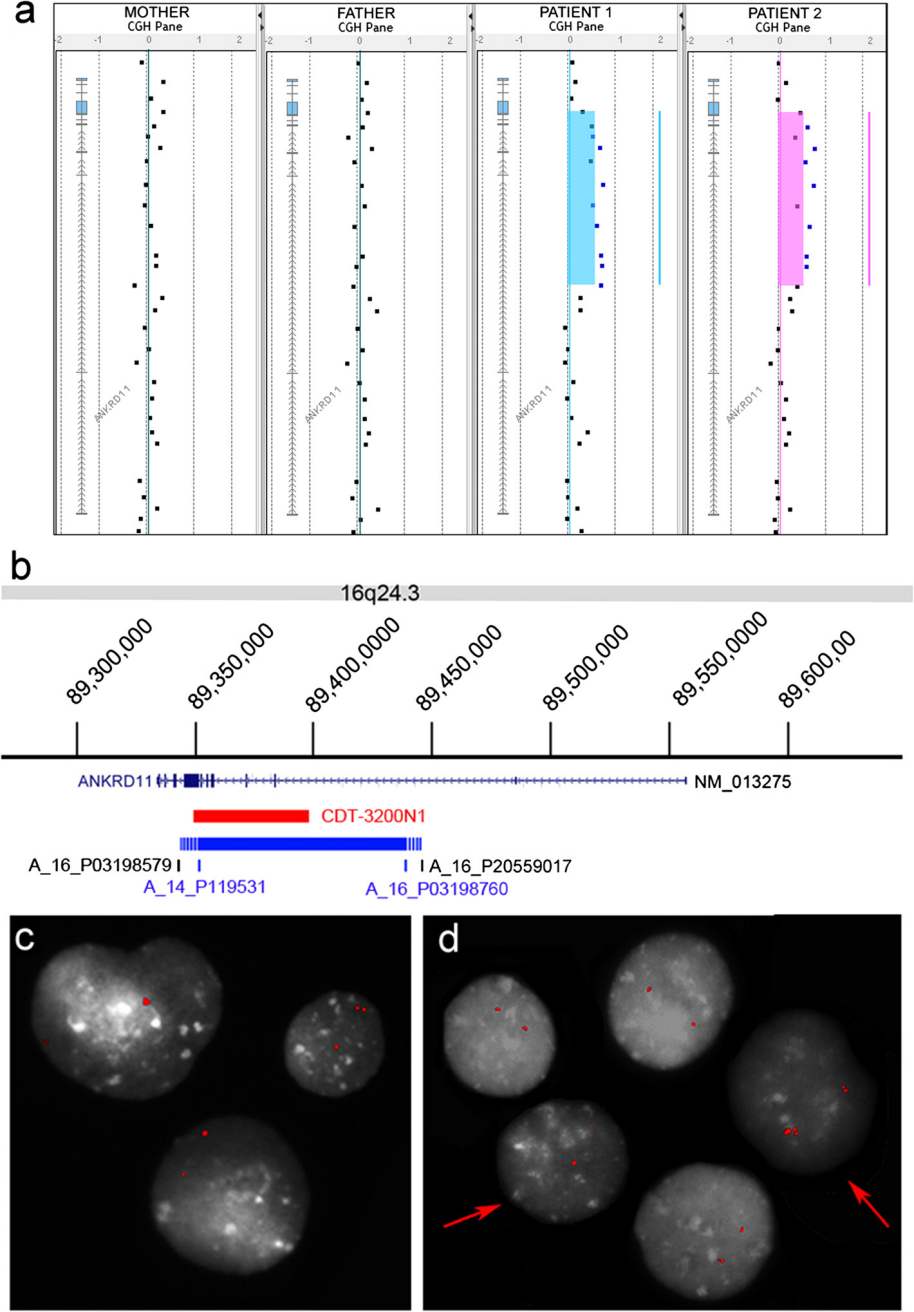
***ANKRD11*** **intragenic duplication in the probands and their mother. (a)** High-resolution array CGH profiles reveal in both sibs a duplication affecting part of the *ANKRD11* coding region. Both parents’ profiles are normal. **(b)** Physical map of 16q24.3 showing the exon/intron structure of *ANKRD11* (dark blue), the BAC probe CTD-3200N1 used in i-FISH (red), the deleted region (blue), and its hypothetical maximum size (dotted line). **(c, d)** i-FISH of peripheral blood lymphocytes from patient 1 **(c)** and his mother **(d)**. BAC probe CTD-3200N1 yields two hybridization signals, one of which is enlarged, in all nuclei of patient 1 **(c)** and in 5% of maternal nuclei **(d)** (red arrow).

Array CGH revealed that both siblings were heterozygous for a novel microduplication of 89 kb in 16q24.3 (chr16:89,350,931–89,439,639, hg19), so far unreported in the Database of Genomic Variants (DGV, http://projects.tcag.ca/variation/project.html) (Figure 2a). The duplication extends from *ANKRD11* intron 2 to exon 9 or intron 10, depending on the minimum and maximum size of the duplication inferred from reference transcript NM_013275.5 (Figure 2b). Thus, the proximal breakpoint occurred between oligonucleotides A_16_P03198579 and A_14_P119531, and the distal breakpoint between oligonucleotides A_16_P03198760 and A_16_P20559017 (Figure 2b). Parental molecular karyotypes were normal (Figure 2a).

Microsatellite polymorphisms at informative loci M1 (chr16:89,391,878-89,391,903, hg19) and M2 (chr16:89,396,404-89,396,438, hg19) revealed the maternal origin of the duplicated allele. Ratios of peak areas for individual alleles showed that both siblings inherited two copies of the maternal allele and one copy of the paternal allele (Additional file 1: Figure S1).

The mother’s attenuated phenotype and microsatellite typing suggested low-degree maternal mosaicism of the duplication; this suspicion was confirmed by i-FISH with BAC probe CTD-3200N1, which partially covers the duplication (Figure 2b). FISH validated the microarray finding, confirming an *ANKRD11* intragenic tandem duplication in both siblings (Figure 2c). Scoring of 500 nuclei in both parents and a normal control revealed that ~5% of maternal cells carried the duplication (Figure 2d); the father exhibited no mosaicism (data not shown).

**Figure 3:**
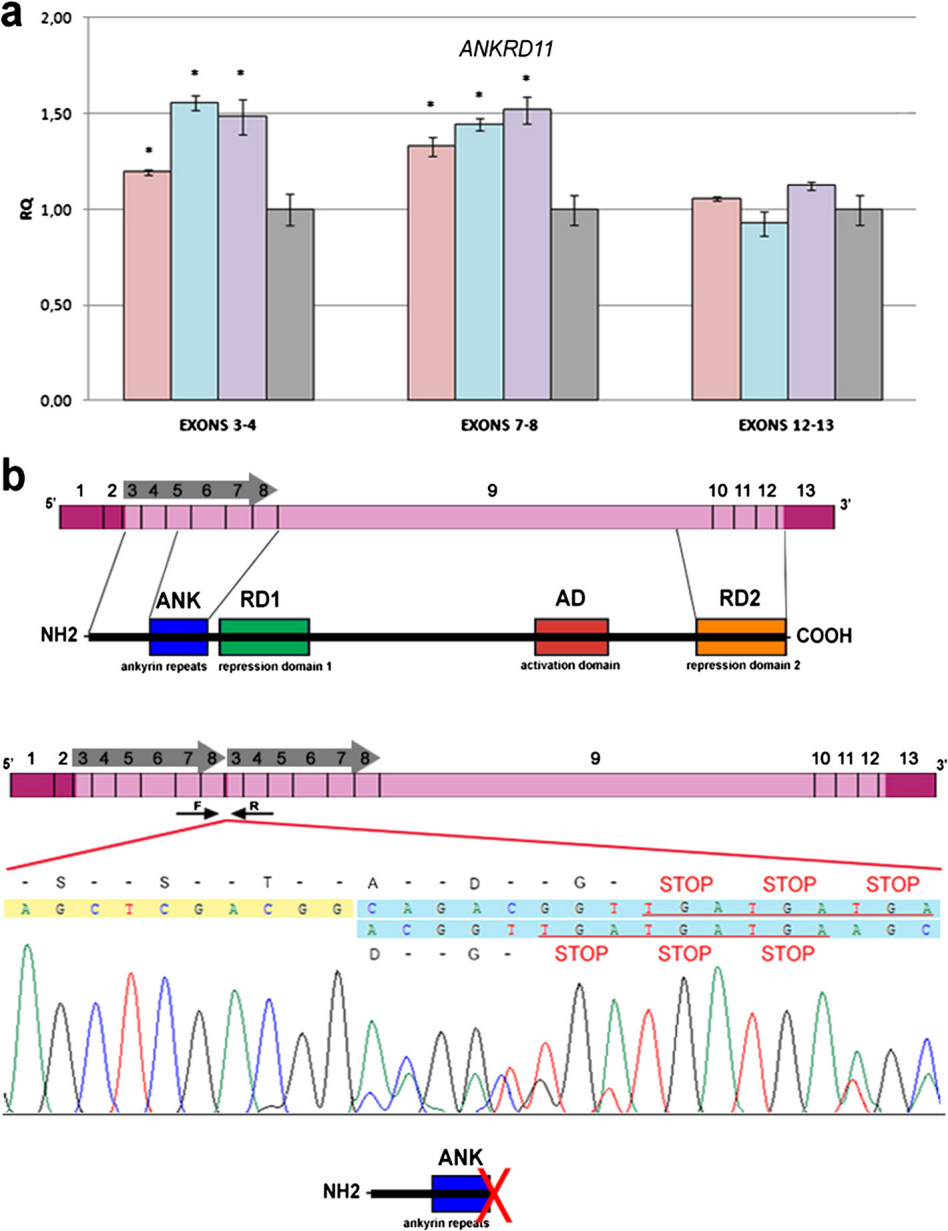
**Molecular characterization of the** ***ANKRD11*** **intragenic duplication. (a)** Relative expression of *ANKRD11* mRNA in lymphocytes of the patients’ mother (pink), male patient (blue), and female patient (lilac), compared to a pool of 10 normal controls (gray), whose value was set to 1, using Taqman probes for exon junctions 2–3, 7–8, and 12–13. *, p < 0.01. **(b)** Graphic representation of wild-type and mutated *ANKRD11* mRNAs and corresponding proteins. Non-coding exons are in pink, and coding exons in light pink. Gray arrows show the duplication of exons 3–8, oriented in tandem. Black arrows indicate positions of primers used to sequence the duplication junction. The electropherogram of the mutant *ANKRD11* transcript is shown, with exon 8 in yellow and exon 3 in blue; the amino-acid sequence is above the nucleotide sequence. Juxtaposition of exon 3 with exon 8 creates a premature termination codon. GenBank accession numbers of the splice-variant transcripts are KM670019 and KM670020. The truncated protein translated from the mutant *ANKRD11* transcript is shown at bottom.

To investigate the pathogenetic effect of the duplication, we quantitated expression of the *ANKRD11* transcript. For exon junctions 3–4 and 7–8, included in the duplicated region, PCR product levels were elevated in probands, whereas for exon junction 12–13, outside the duplication, PCR product levels were comparable in probands and controls (Figure 3a). Consistent with this, direct sequencing of *ANKRD11* cDNA, using primers at the end of exon 8 and the beginning of exon 3, revealed two mutant *ANKRD11* transcripts (Figure 3b). The duplication produces a splice site and so two splice variants were present, one with exon 8 sequence followed by the duplicated exon 3, and the other with deletion of the first codon of the duplicated exon 3 (CAG) (Figure 3b). In both variants, juxtaposition of exon 3 with exon 8 caused a frameshift resulting in premature termination (Figure 3b). The resultant hypothetical truncated protein contains only the ankyrin repeats (Figure 3b).

## Discussion

To date, only point mutations and chromosomal deletions of *ANKRD11* have been reported in patients with KBG syndrome [5-11,13-15]. Here, we describe the first reported case of KBG syndrome with intragenic microduplication of *ANKRD11*. The tandemly duplicated segment extends from intron 2 to exon 9. cDNA sequencing showed the presence of two mutant transcripts, suggesting that the mutant allele is not subject to nonsense-mediated RNA decay. The stability of the mutant transcripts has previously been observed [16]. The rearrangement of our siblings gives rise to a splice site, generating two transcripts, where a frameshift creates a stop codon after exon 8. The predicted truncated ANKRD11 protein should contain the ankyrin repeats (N-terminus) and lack all transcriptional regulatory domains (C-terminus). Notably, point mutations of *ANKRD11* associated with KBG syndrome map to exon 9 or IVS10 and produce similarly truncated proteins [5,16].

Interestingly the rearrangement is present in low-level mosaicism in the probands’ mother, thus documenting a further case of vertical transmission of KBG syndrome [9,14,15]. Parental low level somatic mosaicism is emerging as a frequent mechanism of recurrence as elucidated in recent studies by the increasing sensitivity of genomic technologies with important implications in recurrence risk for couples with children affected by genomic disorders [20]. Consistent with the presence of the microduplication in only 5% of peripheral blood cells, the mother exhibits very mild KBG phenotypes, characterised only by facial dysmorphisms and nasal voice. Thus, as previously postulated [9,16], the phenotypic effect of mosaic *ANKRD11* haploinsufficiency might be dose-dependent.

In addition to typical symptoms of KBG syndrome, both sibling presented with congenital atrioventricular canal defects, a minor feature of KBG syndrome [2,13,15,21]. Our patients also presented with kidney abnormalities not previously reported in KBG patients; to date is difficult to assess if they represent just an association or a further delineation of the known KBG syndrome phenotype.

## Conclusions

Our study discloses a novel pathogenetic rearrangement that triggers *ANKRD11* haploinsufficiency, the same mechanism by which deletions and point mutations cause KBG syndrome. These observations further confirm that *ANKRD11* haploinsufficiency is the cause of KBG syndrome. Molecular evidences demonstrated the pathogenetic effect of the chromosomal duplication identified by array CGH, and led to FISH identification in the mother of the chromosomal aberration overlooked by array CGH due to the low mosaicism level.

## Consent

Written informed consent was obtained from the patients for publication of this Case report and any accompanying images. A copy of the written consent is available for review by the Editor-in-Chief of this journal.

## Additional file

Additional file 1: Figure S1. Identification of the parental origin of *ANKRD11* intragenic duplication by microsatellite analysis. Ratios of peak areas for individual alleles at informative loci, namely M1 (chr16:89391878–89391903, hg19) and M2 (chr16:89396404–89396438, hg19), which both map within the duplication, reveal that both siblings inherited two copies of the maternal allele and one copy of the paternal allele.

## Abbreviations

Array CGH: Array comparative genomic hybridization
DGV: Database of Genomic Variants
i-FISH: Fluorescence in situ hybridization
INeS: Italian Neonatal Study
IUGR: Intrauterine growth restriction
OFC: Occipito-frontal circumference
PCR: Polymerase chain reaction
RT-PCR: Reverse transcription quantitative PCR
SD: Standard deviation
