# Supplementary figures and images for: Familial intragenic duplication of ANKRD11 underlying three patients of KBG syndrome
Source: Mol Cytogenet. 2015 Mar 26;8:20. doi: 10.1186/s13039-015-0126-7 (PMC4383199; doi:10.1186/s13039-015-0126-7)

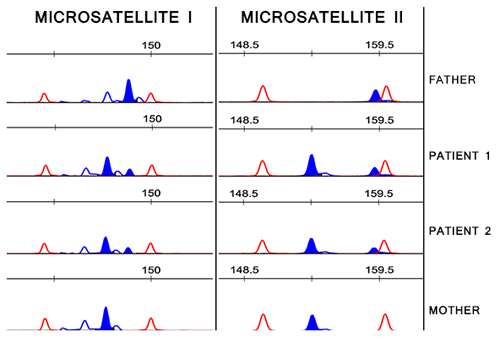

Supplement: Additional file 1: Figure S1. — Identification of the parental origin of ANKRD11 intragenic duplication by microsatellite analysis. Ratios of peak areas for individual alleles at informative loci, namely M1 (chr16:89391878–89391903, hg19) and M2 (chr16:89396404–89396438, hg19), which both map within the duplication, reveal that both siblings inherited two copies of the maternal allele and one copy of the paternal allele. [file 13039_2015_126_MOESM1_ESM.jpeg]
